# Vulnerability of larval and juvenile white sturgeon to barotrauma: can they handle the pressure?

**DOI:** 10.1093/conphys/cot019

**Published:** 2013-07-11

**Authors:** Richard S. Brown, Katrina V. Cook, Brett D. Pflugrath, Latricia L. Rozeboom, Rachelle C. Johnson, Jason G. McLellan, Timothy J. Linley, Yong Gao, Lee J. Baumgartner, Frederick E. Dowell, Erin A. Miller, Timothy A. White

**Affiliations:** 1 Pacific Northwest National Laboratory, Ecology Group, Richland, WA, USA; 2 Confederated Tribes of the Colville Reservation, Spokane, WA, USA; 3 Chinese Sturgeon Research Institute, China Three Gorges Corporation, Yichang, Hubei Province, China; 4 Narrandera Fisheries Centre, Department of Primary Industries, Narrandera, NSW, Australia; 5 Pacific Northwest National Laboratory, Radiation Detection and Nuclear Sciences Group, Richland, WA, USA

**Keywords:** Barotrauma, hydropower, hydroturbine, larvae, swim bladder, white sturgeon

## Abstract

Techniques were developed to determine which life stages of fish are vulnerable to barotrauma from expansion of internal gases during decompression. Eggs, larvae, and juvenile hatchery-reared white sturgeon (*Acipenser transmontanus*; up to 91 days post hatch; d.p.h.) were decompressed to assess vulnerability to barotrauma and identify initial swim bladder inflation. Barotrauma-related injury and mortality were first observed 9 d.p.h., on the same day as initial exogenous feeding. However, barotrauma-related injury did not occur again until swim bladder inflation 75 d.p.h. (visible at necropsy and on radiographs). Swim bladder inflation was not consistent among individuals, with only 44% being inflated 91 d.p.h. Additionally, swim bladder inflation did not appear to be size dependent among fish ranging in total length from 61 to 153 mm at 91 d.p.h. The use of a combination of decompression tests and radiography was validated as a method to determine initial swim bladder inflation and vulnerability to barotrauma. Extending these techniques to other species and life-history stages would help to determine the susceptibility of fish to hydro turbine passage and aid in fish conservation.

## Abstract (precis)

Techniques were developed to determine when fish are vulnerable to barotrauma when rapidly decompressed during hydroturbine passage. Sturgeons were decompressed in early life-stages and X-ray radiographs were taken to determine when gas was present in the swim bladder. Barotrauma was observed on day 9 and greater than 75 days after hatching.

## Introduction

Studies of fish passage through hydro structures have primarily focused on actively migrating life stages; however, little is known of the effects on passively drifting egg or larval life stages, which have a high probability of interaction with hydro structures. This issue may be cumulative with the addition of new hydro structures and the upgrading of existing hydro structures. In the forthcoming decades, global human populations are expected to expand rapidly (Cooke *et al.*, 2013), leading to a global expansion of new water resource infrastructure (Rosegrant *et al.*, 2002), such as hydroelectric dams and irrigation weirs (Baumgartner *et al.*, 2012). Within the USA, the Department of Energy has a goal of adding hydroturbines to existing hydro projects to expand energy output (Hadjerioua *et al.*, 2012). These types of activities will increase the likelihood that fish will interact with hydro structures. Thus, it is important to understand how fish of all life stages may be influenced by these changes, in order to to guide conservation measures.

When moving downstream through hydro structures, fish pass via spillways, turbines, bypass facilities, or weir gates, all of which expose fish to varying degrees of pressure changes. Turbine passage is considered the most harmful to fish due to the possibility of also encountering injuries from blade strike and shear forces (Deng *et al.*, 2005, 2007, 2010; Čada *et al.*, 2006; Brown *et al.*, 2009, 2012a, b). Blade strike susceptibility is largely proportional to body size (Franke *et al.*, 1997), and not all fish will be exposed to damaging levels of shear (Deng *et al.*, 2007). However, all fish are exposed to rapid pressure changes (Deng *et al.* 2010).

When fish are exposed to rapid changes in pressure, they are vulnerable to barotrauma, which is injury associated with decompression (Brown *et al.*, 2009). Damage due to barotrauma can include emboli in the gills, haemorrhage, ruptured swim bladder, and exophthalmia. Brown *et al.* (2012b) found that barotrauma in juvenile salmonids passing hydroturbines was largely due to the expansion of gas within the swim bladder, the rupture of the swim bladder, and damage caused when escaping gas causes haemorrhaging, emboli, and exophthalmia. Therefore, the presence, form, function, and type of a swim bladder, whether it is inflated or not, and the amount of gas within it can influence the vulnerability of fish to barotrauma. Swim bladders can be either open (physostomous) or closed (physoclistous). Open swim bladders contain a connection with the oesophagus, meaning that the fish can regulate swim bladder volume, hence buoyancy, by either gulping or expelling gas. Physoclistous fish regulate swim bladder volume through an organ known as the rete mirabile, which regulates volume by exchanging gas from the blood. The presence and design of a swim bladder can greatly influence susceptibility to barotrauma. For example, lamprey (juvenile brook and Pacific; *Lampetra richardonii* and *Entosphenus tridentatus*, respectively), which do not have a swim bladder, were uninjured when exposed to extreme pressure change scenarios known to cause injuries and mortalities in juvenile Chinook salmon, a physostomous species (Colotelo *et al.*, 2012). Physostomous species, such as white sturgeon and salmon, can evacuate gas from the swim bladder and expel it from the mouth and gill covers, which can make them less vulnerable to barotrauma than physoclists. For example, bluegill (*Lepomis macrochirus*) have been shown to have greater injury and mortality than physostomous, juvenile Chinook salmon, when exposed to similar rapid decompression scenarios (Abernethy *et al.*, 2001).

Expansion of gas in the swim bladder is governed by Boyle's Law (Van Heuvelen, 1982), which states that the volume of gas is inversely proportional to the pressure acting on the volume. Thus, if the surrounding pressure is decreased by half, the volume of pre-existing gas in the swim bladder will double. Vulnerability to barotrauma is therefore dependent on the amount of gas in the swim bladder, which is dependent, among other factors (e.g. position in the water column; Pflugrath *et al.*, 2012), on the life stage of the fish (Tsvetkov *et al.*, 1972).

In most fish species, there is little known regarding the timing of the initial inflation of the swim bladder, which may mark the time in the fish's life when they are first vulnerable to barotrauma due to pressure changes when passing hydro structures. The time of initial swim bladder inflation is variable and can be dependent on the age of the fish, the water temperature, and the rearing environment (Harrell *et al.*, 2002). Initial swim bladder inflation has also been found to vary greatly among species. Swim bladder inflation in larval rainbow trout (*Oncorhynchus mykiss*) was documented to occur at around 24 days post hatch (d.p.h.; Tait, 1960), while it can occur as early as 4–5 d.p.h. in zebrafish (*Danio rerio*; Çelik *et al.*, 2011), and 7–14 d.p.h. in walleye (*Stizostedion vitreum*; McElman and Balon, 1979).

Susceptibility to barotrauma is less likely to be a major concern among life stages of fish that have demersal egg and larval stages, because these are not likely to encounter hydro structures. However, in fish species with passively drifting eggs or larvae, these can travel long distances and are therefore likely to interact with hydro facilities. Several fish species with drifting larvae in North America include sturgeon (*Acipenser* spp.), paddlefish (*Polyodon spathula*), and walleye (*Sander vitreus*). Sturgeon are especially important to study, because many populations have become reduced from historical levels and are still declining (Ptolemy and Vennesland, 2003), and many inhabit impounded river systems (e.g. the Columbia River in the Pacific Northwest, USA). Larval white sturgeon (*A. transmontanus*) may passively drift great distances from spawning grounds (McCabe and Tracy, 1994). Additionally, some studies have observed a secondary active stage of downstream dispersal in juvenile white sturgeon and other species. However, the timing and duration of this phase are variable among species and not well studied (Utter *et al.*, 1985; Kynard and Parker, 2005; Kynard *et al.*, 2010).

Developing larval and small juvenile fish may be more vulnerable to rupture of the swim bladder during decompression if swim bladders are not robust (Tsvetkov *et al.*, 1972). Furthermore, small larvae may not be effectively diverted around hydroturbines by screen systems established for larger fish. In southeast Australia, for example, high mortality among drifting larval Murray cod (*Maccullochella peelii*) and golden perch (*Macquaria ambigua*) has been found when passing irrigation weirs (Baumgartner *et al.*, 2006). Thus, there remains a concern that species with drifting larvae are particularly vulnerable, especially if they have developed and inflated swim bladders.

The goal of this research was to develop techniques to determine at which early life stages white sturgeon may be vulnerable to barotrauma. Previous research has indicated that the presence of undissolved gas within the body of a fish, such as the air in an inflated swim bladder (although the type of gas within the swim bladder can vary with fish types; see Fänge, 1983), is a key determinant of vulnerability to barotrauma during simulated hydro turbine passage (Brown *et al.*, 2012b). Therefore, determining if or when undissolved gas is present and when initial inflation of the swim bladder occurs is essential information for understanding the vulnerability to barotrauma among fish passing hydro structures. Better understanding of the physiology of sturgeon can provide a broader understanding of how humans can impact these populations. Linking an understanding of physiological pathways can thus aid in the protection and conservation of fish, as was brought to light by Cooke *et al.* (2013).

Decompression experiments were used to determine when white sturgeon first filled their swim bladders. We predicted that when decompressed, any undissolved gas would expand, causing fish to float or to expel gas from their swim bladder, and appear as bubbles coming out of the mouth or gill covers, providing an indication of swim bladder inflation. In addition, similar to several studies (Lundin and Holmgren, 1991; Barrows *et al.*, 1993; Robertson *et al.*, 2008; Brown *et al.*, 2012b; Geist *et al.*, 2013), we used radiography to confirm the presence of gas within their bodies.

## Materials and methods

On 10 July 2012, ∼20 000 fertilized white sturgeon eggs were obtained from Sherman Creek Hatchery, Kettle Falls, WA, USA. The eggs were transported to the Pacific Northwest National Laboratory Aquatic Research Laboratory in Richland, WA, USA for incubation and rearing. Eggs were incubated in MacDonald upwell jars supplied with flow-through Columbia River water at 14°C (±2°C). The larvae hatched between 6 and 8 days after fertilization (16–18 July 2012). After 2 days of hatching, all remaining eggs were removed to ensure a known fish age within ∼2 days. As such, days post hatch should be considered as *x*th day ±1 throughout. Hatched larvae were held in rectangular rearing troughs (197 l raceways; 0.3 m × 0.3 m × 3.0 m) supplied with 14–18°C flow-through Columbia River water. Starting at 9 d.p.h., fish were fed Otohime Marine weaning diets and freeze-dried copepods.

### Description of the pressure system

All testing was conducted in hyper/hypobaric chambers, described by Brown *et al.* (2009). The system consisted of two 27.5-cm-diameter acrylic tubes, 55 cm long, with a volume of 34 l each. Hydraulic pistons were moved to modify pressures within the chambers. The hydraulic pistons were moved by pneumatic pressure and controlled manually by manipulating air-supply valves. During this study, the chambers were supplied with 14–18°C flow-through Columbia River water.

### Exposure to pressure reductions

Pressure reduction is expressed in terms of a ratio (similar to Brown *et al.*, 2012a). This ratio of pressure change (RPC) is the pressure of acclimation (where the fish is neutrally buoyant if they have a swim bladder) divided by the lowest pressure of the exposure (termed the nadir). Prior to exposure, the eggs, larvae, and juveniles were acclimated in the chambers at surface pressure (101 kPa) for 1 h. A bubble was left at the top of the chambers to allow the fish to fill their swim bladder by gulping air at the surface. This behaviour of gulping air at the surface has been observed previously in juvenile salmon (Harvey, 1963). Decompression from surface pressure occurred over ∼2–6 s. The RPC to which the fish were exposed ranged from 2.6 to 21.0 (nadirs of 5–39 kPa), which would lead to undissolved gas within the body of the fish expanding to 2.6–21.0 times its original volume during decompression. These pressure exposures were not meant to simulate turbine passage but to ensure that gas expansion occurred.

Pressure testing began with the eggs. Eggs were transferred with a pipette from the incubation jars to a glass beaker. The beaker was filled with water and placed directly into the empty pressure chamber. The pressure chamber was then filled with water, and then eggs were decompressed. Immediately following exposure, eggs were retrieved from the chamber and photographed with a microscope camera. Eggs were decompressed after 3 and 7 days of incubation (*n* = 17 and 25, respectively, in each group).

Sturgeon larvae and juveniles were decompressed every second day from 2 until 42 d.p.h. Given that the initial swim bladder filling took longer than anticipated, sampling was reduced to a minimum of once a week between 41 and 76 d.p.h. On each sampling day, at least 10 fish were exposed to pressure reductions. During acclimation and exposure, fish were observed and recorded with a security camera system (CCTV Security Pros, 16 Camera Pro Series, CSP-16PRO650IR).

Upon completion of the pressure exposure, fish were removed from the chamber, observed for any erratic swimming or mortality, and euthanized with buffered 250 mg/l tricaine methanesulfonate (MS-222; Finquel; Argent Chemical Laboratories, Redmond, WA, USA). Radiographs of exposed and unexposed fish were taken periodically (at 7, 10, 17, 23, 38, 78, and 91 d.p.h.) to check for the presence of gas and any abnormalities in exposed fish. Radiographs (for more detail on radiography, refer to Barrett and Swindell, 1981) were taken using a tungsten anode tube source (MXR-160HP/11; Comet, Stamford, CT, USA) operated at 40 kV 2 m upstream of the sample. A digital detector (Shad-o-box 4k; Teledyne Dalsa Inc., Waterloo, ON, Canada) was placed immediately behind the sample for an effective pixel size of 50 μm. Images shown indicate the fraction of the original beam intensity reaching the detector. Low-density areas, such as undissolved gasses, result in higher intensities and are visible as lighter areas.

## Results

There was no indication that undissolved gas was present within eggs when examined visually under the microscope. When placed in a beaker to be tested, eggs were negatively buoyant and rested on the bottom. During decompression, there was no indication of the eggs becoming positively buoyant; the eggs all remained on the bottom of the beaker.

During the first 8 d.p.h., there was no indication of barotrauma or swim bladder inflation among the larval sturgeon. No fish became positively buoyant (floated) when decompressed, and no emboli, haemorrhaging, or exophthalmia were externally visible. Also, no evacuation of gas from the swim bladder was observed exiting the mouth, gill covers, or vent of the fish, and they did not swim erratically when decompressed.

**Figure 1:**
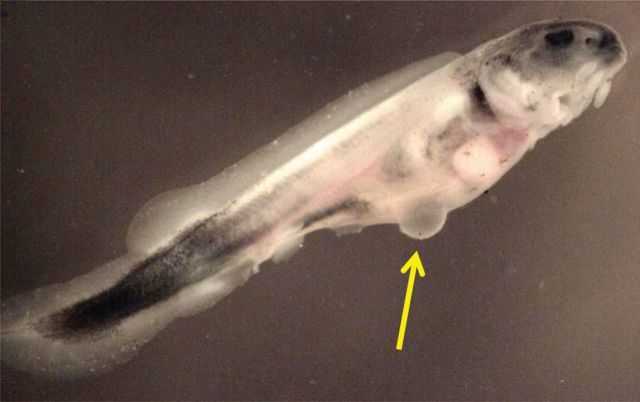
A herniation-like injury (indicated by the arrow) in the abdomen of a larval sturgeon after decompression, 9 days post hatch.

**Figure 2:**
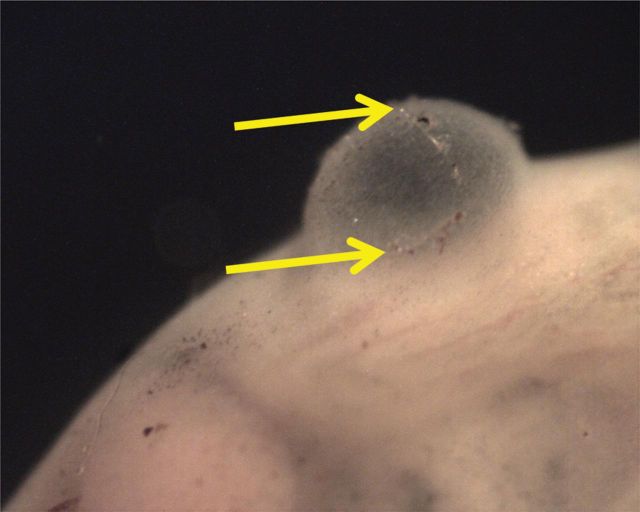
A possible tear (indicated between the two arrows) on a herniation-like injury in the abdomen of a larval sturgeon after decompression, 9 days post hatch.

On the same day that fish were first fed (9 d.p.h.), injuries were seen among decompressed fish. In the first of two exposures tested that day, one of the 10 fish (10%) swam erratically and died within 10 min of the exposure (RPC = 2.7). Several of the other fish in this exposure appeared agitated. Due to the mortality and agitated behaviour, a second exposure of additional fish was completed. In the second exposure (RPC = 5.5), one fish was dead within a few minutes of exposure. Two other fish were swimming erratically within a few minutes of exposure, and one of these died within 15 min of exposure while the other recovered. Among the two exposures, 15% of the fish died (10% in the first exposure and 20% in the second). Other injuries were also observed; the dead fish from the first exposure expelled a globule of a yellow lipid-like substance when decompressed. There was also the presence of a herniation-like abnormality in the abdomen (Fig. 1) of the fish that was dead within a few minutes of decompression (in the second trial). It appeared as though there was a possible tear in the herniation-like injury (Fig. 2).

### Indications of swim bladder inflation

From 10 to 75 d.p.h., we saw no further indication of barotrauma injury due to decompression and also no indication of swim bladder inflation. There were no external signs of emboli, haemorrhaging, or exophthalmia and no erratic swimming behaviour. There were also no apparent differences in fish behaviour related to the different degrees of decompression.

At 75 d.p.h., several fish expelled gas from their mouths when decompressed (RPC 5.4), providing evidence that some fish had filled their swim bladder. Among the seven fish examined (mean length 76.6 mm, range 46–115 mm; and mean weight 2.2 g, range 0.4–5.5 g), gas was visible in the swim bladder in the radiographs of five individuals. The presence of gas did not appear to be dependent upon the size of the fish. The two fish that did not have gas in their swim bladder were 48 and 94 mm long.

**Figure 3:**
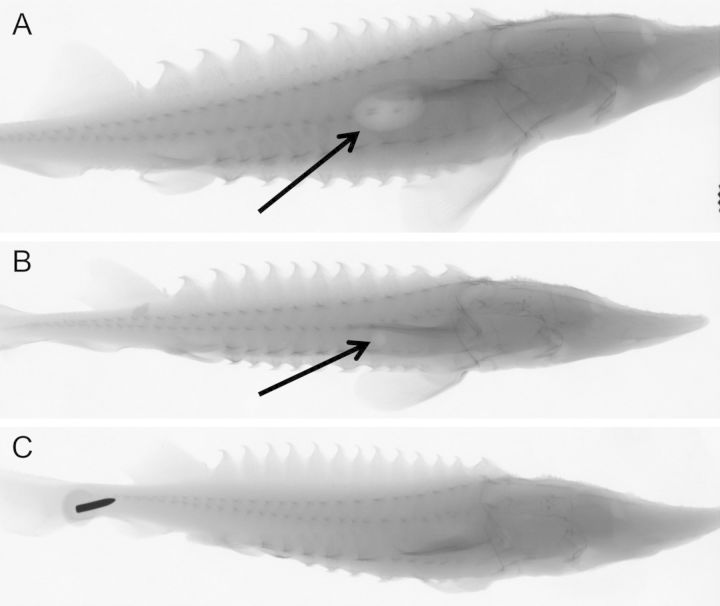
Radiographs of juvenile white sturgeon showing varying degrees of swim bladder inflation 91 days post hatch. Fish pictured have swim bladders largely inflated (**A**; swim bladder indicated by arrow; 122 mm long), minimally inflated (**B**; 109 mm long), and non-inflated (**C**; 87 mm long). The swim bladder is indicated by a light-coloured area because it is low in density. The black arrow near the caudal peduncle of the fish in C is a tack, which is holding the fish to a panel perpendicular in front of the X-ray machine.

Once it was confirmed that swim bladder inflation had occurred, a final set of radiographs were acquired at 91 d.p.h. on 24 fish to evaluate the percentage of fish with inflated swim bladders. Of these 24 fish, six were exposed to decompression to assess potential effects of barotrauma after swim bladder inflation. Among the 18 fish not decompressed, eight (44%; ranging in length from 66 to 153 mm) appeared to have gas in their swim bladder while 10 (56%; ranging in length from 61 to 139 mm) did not. There was also large variation in the relative amount of gas in the swim bladder among individuals that did not seem to be influenced by fish size (Fig. 3).

**Figure 4:**
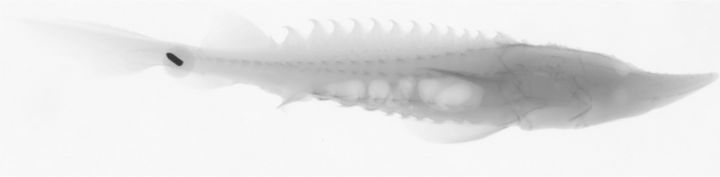
Radiograph of an 88-mm-long juvenile sturgeon with gas in its body cavity and intestinal tract following decompression (91 days post hatch). The bubbles are indicated by light-coloured areas because they are low in density. The black arrow near the caudal peduncle of the fish is a tack, which is holding the fish to a panel perpendicular in front of the X-ray machine.

**Figure 5:**
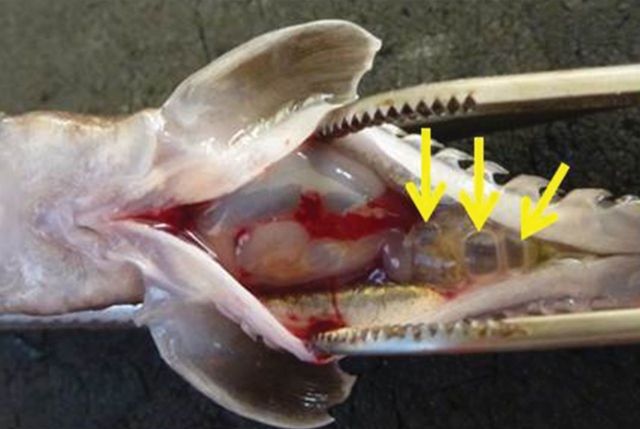
An image of a juvenile sturgeon with gas in its body cavity and intestinal tract following decompression (91 days post hatch).

Of the six fish that were decompressed prior to radiography, four individuals appeared to have gas within their swim bladders. The radiographs also showed that half of these fish had bubbles outside of the swim bladder in the intestines, within the body cavity, or both (Fig. 4). Necropsies confirmed the presence of bubbles present within the intestines (Fig. 5). Erratic swimming was also noted among some of the decompressed fish.

### Indications of metabolic gas

Several weeks (205 d.p.h.) after the completion of barotrauma testing, gas was noted in the intestine of a juvenile sturgeon that had not been exposed to pressure changes. This gas appeared as small bubbles throughout the intestine.

## Discussion

White sturgeon inflated their swim bladders in the juvenile stage. Specifically, we observed swim bladder inflation to occur first at ∼75 d.p.h. White sturgeon may, however, be vulnerable to barotrauma as early as the larval stage. The results suggest that they may be vulnerable to barotrauma at two life stages, i.e. as larvae at 9 d.p.h., when initial feeding occurred, and as small juveniles once they began filling their swim bladders. During this research, we also validated the use of decompression experiments coupled with radiographic imaging to confirm the initial inflation of swim bladders in developing fish. Although these results are only a beginning to the understanding of the effects of hydro structure passage on drifting larvae and/or freely swimming juveniles, this work serves to highlight the many knowledge gaps still present in this area of research.

The purpose of this work was to determine the initial inflation of the swim bladder through decompressions; the exposure pressures were not meant to simulate real passage scenarios through structures such as hydro turbines. Study fish were exposed to relatively low pressures in order to ensure that even small amounts of pre-existing gas (such as in the swim bladder) would expand. Although pressure reductions of this scale may occur during turbine passage, pressures between 50 kPa and surface pressures (101 kPa) are likely to be more common (Deng *et al.*, 2007, 2010; Carlson *et al.*, 2008). We therefore suggest that further research should be done to understand more about the relationship between pressure change and barotrauma along a broad range of pressure changes and rates of pressure change, similar to research done on Chinook salmon by Brown *et al.* (2012a, c).

We were unable to identify published literature on the developmental biology of the swim bladder in sturgeon, making it difficult to assess the probability of injury during hydro passage. For example, sturgeon may experience higher ratios of pressure change if they have an active rete (the bed of vasculature that transfers gas from the blood into and out of the swim bladder; Fänge, 1983). Having an active rete would allow them to become neutrally buoyant at greater depths. This could equate to a greater amount of gas in the swim bladder, which would expand dramatically if the fish were exposed to hydro turbine passage (Čada, 1990; Brown *et al.*, 2012b). Understanding this topic further is essential to the interpretation of some of our observations.

Most of the barotrauma research conducted on freshwater fish to date has focused on juvenile salmonids, which typically pass several hydroelectric dams when making their seaward migrations (McMichael *et al.*, 2010). As such, a comprehensive understanding of swim bladder function exists for some juvenile salmonids, and their ability to vent gas from the pneumatic duct upon decompression has been confirmed (Brown *et al.*, 2012b). However, the form of the swim bladder in non-salmonid species appears to be different (Fänge, 1983). The pneumatic duct of white sturgeon is present at the ventral part of the swim bladder instead of on the anterior part of the swim bladder as it is in salmonids. This may lead to escaping gas entering into the intestines, as was noted during the present research; it is not clear where else these large volumes of gas could have originated.

Barotrauma was first observed when study fish began exogenous feeding. It remains unknown whether this was an artifact of the laboratory setting (i.e. pellets vs. natural food sources) or if the same degree of mortality and injury would occur with more realistic (i.e. closer to surface pressure) decompression scenarios. These results do, however, raise concern for this life stage and emphasize the need to determine how much gas may be produced by metabolic processes once larval sturgeon or other fish begin feeding. Bubbles of such gas in the intestinal tract could have caused the herniation-like injury or the expulsion of yellow fluid observed. Given that the fish was exposed to an RPC of 5.5, even a small bubble would increase in size to a great degree (by 5.5 times its original volume), possibly causing this injury. Observations of gas bubbles in the intestines 205 d.p.h. indicate that metabolically produced gas could be of concern in barotrauma research.

While little research has been done on the volume of gas produced by metabolic processes in the gastrointestinal tract of fish, there have been investigations into the microflora of sturgeon and other species (Trust *et al.*, 1979; Callman and Macy, 1984). The metabolisms of these intestinal bacteria could be sources of undissolved gases in the gastrointestinal tract. Within the fish species that have been studied regarding gastrointestinal bacteria, there is a predominance of anaerobic bacteria over strictly aerobic bacteria (Trust *et al.*, 1979; Skrodenyte-Arbaciauskiene, 2007; Skrodenyte-Arbaciauskiene *et al.*, 2008). Some common products of anaerobic fermentation processes are gases, such as methane and hydrogen gas.

The predominant anaerobic species in the intestines of white sturgeon, a *Bacteroides* spp., has been shown to produce H_2_ (Callman and Macy, 1984). Callman and Macy (1984) also confirmed that H_2_ is present, and is the only detectable gas within the intestine and the swim bladder of white sturgeon (although nitrogen gas could not be detected with their methods). These gases of intestinal origin in sturgeon are of special interest, because the pneumatic duct is close to the hindgut. The production of this gas as a result of nutrients entering the gastrointestinal tract could explain the first signs of barotrauma observed after the first exogenous feeding. Thus, it is possible that gas produced in the gut could enter the swim bladder.

The second observed occurrence of barotrauma was during the juvenile stage, once swim bladder inflation was confirmed. However, sturgeon in the wild may fill their swim bladders at a different time than those raised in fish culture conditions. Given that the holding tanks were quite shallow, the need to fill the swim bladder and regulate buoyancy may not be as important as for those fish developing in deeper riverine environments.

The probability of encountering hydropower structures during the juvenile stage is unclear, because there is a lack of research on fish that are in the earlier phases of life. While it is common for telemetry research to be conducted on relatively large juvenile sturgeon (>250 mm fork length; e.g. Geist *et al.*, 2005; Golder Associates, 2006; Parsley *et al.*, 2008, 2011; Neufeld and Rust, 2009), movements of early life-stage white sturgeon have received little focus. The long-distance movements reportedly achieved by early life-stage white sturgeon were based on captures downstream of known spawning areas, rather than tracking of real movements (McCabe and Tracy, 1994; Howell and McLellan, 2008, 2011). The small size (<100 mm fork length) of early life-stage white sturgeon limits the ability to study their movements using telemetry. Typically, larval sturgeon are sampled using trawls and plankton nets (Parsley *et al.*, 1993; McCabe and Tracy, 1994; Howell and McLellan, 2011), but information on larger juveniles is less available, possibly owing to an ability to avoid these gears. Passive integrated transponder tags have been injected into sub-yearling hatchery-reared white sturgeon as small as 6 g (mean fork length 103 mm; Washington Department of Fish and Wildlife, unpublished data), but most juvenile white sturgeon that have been passive integrated transponder tagged have been larger (e.g. >175 mm fork length and ≥5 months post-hatch; Golder Associates, 2007; Justice *et al.*, 2009; Howell and McLellan, 2011). Movement information has been derived from recapture of passive integrated transponder-tagged juvenile white sturgeon, but is sparse due to the low numbers of recaptures of individual fish and the relatively long periods of time (months or years) between recaptures. A lack of information concerning white sturgeon early life-stage movements impedes our ability to assess the risk of barotrauma in the present river environment. There is therefore a need for research to be conducted on smaller transmitters that could be used to gather information on young sturgeon (in the first year of their life) to determine the likelihood that they are passing hydro structures and are being exposed to pressure reductions that could lead to barotrauma.

Additionally, it would be beneficial to use the techniques detailed in the present research to determine the vulnerability to barotrauma of eggs, larval, and small juveniles of other fish species and aid in their conservation. This is especially the case in Australia and in tropical rivers, where there are many species with a larval drift phase that may make interactions between fish larvae and hydro facilities commonplace (Baumgartner *et al.*, 2006).

In conclusion, we suggest that further work should be focused on determining how vulnerable the relatively fragile, larval and small juvenile fish are to barotrauma due to proximity to hydropower and other water-management structures. This should include relating mortality and injury in sturgeon to exposure to a broad range of pressure changes. Use of the techniques outlined in the present research could provide valuable data for conserving species with drifting eggs, larvae, or small juveniles. In addition, we suggest that further research should be conducted to examine the presence of metabolically produced gas in fish, and how its presence may lead to barotrauma during hydro turbine passage.
